# Combination therapy with fosfomycin for *Staphylococcus aureus* bacteraemia or endocarditis: a systematic review and meta-analysis of randomized trials

**DOI:** 10.1093/jacamr/dlaf101

**Published:** 2025-06-17

**Authors:** Ahmad Mourad, Joshua B Parsons, Lesley A Skalla, Thomas L Holland, Timothy C Jenkins

**Affiliations:** Department of Medicine, Division of Infectious Diseases, Duke University Medical Center, Durham, NC, USA; Duke Clinical Research Institute, Duke University School of Medicine, Durham, NC, USA; Department of Medicine, Division of Infectious Diseases, Duke University Medical Center, Durham, NC, USA; Duke University Medical Center Library & Archives, Duke University School of Medicine, Durham, NC, USA; Department of Medicine, Division of Infectious Diseases, Duke University Medical Center, Durham, NC, USA; Duke Clinical Research Institute, Duke University School of Medicine, Durham, NC, USA; Division of Infectious Diseases, Department of Medicine, Denver Health, Denver, CO, USA; University of Colorado School of Medicine, Aurora, CO, USA

## Abstract

**Background and objectives:**

Fosfomycin combination therapy for *Staphylococcus aureus* bacteraemia or endocarditis has been evaluated, but studies were limited by small sample sizes. We sought to conduct a systematic review and meta-analysis of randomized controlled trials (RCTs) to establish robust efficacy and safety estimates of fosfomycin combination therapy in this patient population.

**Data sources:**

MEDLINE, Embase, Cochrane Library and Web of Science databases were searched from inception through September 2024 (PROSPERO CRD42024583822).

**Study eligibility:**

RCTs comparing fosfomycin combination with standard-of-care antibiotics in patients with *S. aureus* bacteraemia or endocarditis were included. Two independent reviewers screened studies for inclusion.

**Assessment of risk of bias:**

Risk of bias was assessed using the revised Cochrane RoB 2 tool.

**Data synthesis and analysis:**

Treatment effects were estimated with pooled risk ratios (RRs) using random effects meta-analysis. Heterogeneity between studies was assessed with Cochran's Q-statistic and *I*^2^ test.

**Results:**

Of 437 articles identified, three RCTs met inclusion criteria. Primary outcome of treatment success or cure was not meta-analysed due to clinical heterogeneity. Combination therapy did not significantly improve mortality (RR 0.85; 95% CI, 0.28–2.52; *I^2^* = 27.8%) or persistent bacteraemia (RR 0.34; 95% CI, 0.04–2.59; *I^2^* = 0%). Participants receiving combination therapy had more adverse events leading to treatment discontinuation, but this was not statistically significant (RR 1.84; 95% CI, 0.36–9.36; *I^2^* = 18%).

**Conclusions:**

In this meta-analysis of three RCTs, fosfomycin combination therapy for *S. aureus* bacteraemia or endocarditis did not significantly improve patient outcomes and may be associated with higher rates of adverse events.

## Introduction

*Staphylococcus aureus* bacteraemia is common and lethal, despite advances in antibiotic therapy and decades of clinical research.^1^ The current standard-of-care antibiotic therapy for patients with *S. aureus* bacteraemia is monotherapy with either vancomycin or daptomycin for MRSA, or an anti-staphylococcal penicillin or cephalosporin for MSSA.^2–5^ However, given that over one-quarter of patients do not survive their infection with this current standard of care,^6,7^ several studies, including randomized controlled trials (RCTs), have explored the role of routine combination antibiotic therapy to improve clinical outcomes.^8^

Fosfomycin, a phosphonic acid antibiotic that inhibits bacterial peptidoglycan synthesis, has potent *in vitro* activity against both MSSA and MRSA, with the ability to penetrate and disrupt biofilms, and demonstrates a synergistic effect when combined with glycopeptides, daptomycin or β-lactams.^9–11^ As such, there has been interest in evaluating the effect of the addition of fosfomycin to standard-of-care antibiotics (hereafter referred to as combination therapy with fosfomycin) on clinical outcomes of patients with invasive *S. aureus* infections. A single-arm study utilizing a regimen of imipenem combined with fosfomycin for patients with MRSA bacteraemia or endocarditis requiring ‘rescue’ therapy, showed that the combination was effective at sterilizing blood cultures and was well tolerated.^12^ However, recent RCTs in patients with *S. aureus* bacteraemia or endocarditis suggest that although combination therapy with fosfomycin may reduce rates of persistent bacteraemia, it may also increase treatment-related adverse events.^13,14^ These studies have been limited by relatively small sample sizes with resultant uncertainty in their conclusions regarding efficacy and safety. We therefore conducted a systematic review and meta-analysis of RCTs to increase statistical power and improve estimates of the efficacy and safety of combination therapy with fosfomycin compared with standard-of-care antibiotic therapy for *S. aureus* bacteraemia or endocarditis.

## Methods

This systematic review was conducted using the Cochrane Handbook for Systematic Reviews of Interventions guidance,^15^ and reported according to the Preferred Reporting Items for Systematic Reviews and Meta-Analyses (PRISMA) guidelines.^16^ The PRISMA-SR checklist is provided in Table S1 (available as Supplementary data at *JAC-AMR* Online). The study protocol was registered in PROSPERO prior to the conduct of the study (CRD42024583822); amendments to relevant endpoint definitions were subsequently added and posted. We sought to answer the clinical question, ‘For patients with bacteraemia or endocarditis caused by *S. aureus*, what is the efficacy and safety of combination therapy with fosfomycin compared with standard-of-care antibiotic therapy?’

### Search strategy

We searched for RCTs published in any language from database inception through September 12, 2024, comparing participants with *S. aureus* bacteraemia or endocarditis treated with fosfomycin combination therapy with those treated with standard-of-care therapy. The search included MEDLINE (via PubMed), Embase (Elsevier), Web of Science Core Collection, All Editions (Clarivate) and the Cochrane Central Register of Controlled Trials (Wiley). The search strategy was developed and implemented by a medical librarian (L.A.S.) and peer reviewed by an additional independent medical librarian using modified Peer Review of Electronic Search Strategies.^17^ The detailed search strategy is shown in Table S2 and can be accessed via https://duke.is/m/kap5. A manual search of references was also conducted to identify studies that were not captured by this search. Articles were imported into Covidence, a web-based platform that facilitates the conduct of systematic reviews, for identification of duplicates and screening.^18^

### Study selection, data extraction and quality assessment

Studies were included in our review if they met the following four pre-specified criteria: (i) the study was an RCT—given the heterogeneity of disease states and patient populations with *S. aureus* bacteraemia and endocarditis, and the potential for introduction of significant confounders in observational studies (i.e. confounding by indication), we only included RCTs in this review; (ii) included participants with *S. aureus* bacteraemia and/or endocarditis and reported their outcomes; (iii) included participants receiving combination antibiotic therapy with fosfomycin for *S. aureus* bacteraemia and/or endocarditis in an intervention arm, and reported their outcomes; and (iv) included participants receiving standard-of-care antibiotic therapy for *S. aureus* bacteraemia and/or endocarditis in a comparator arm, and reported their outcomes.

Screening of titles and abstracts, and full-text review for inclusion of studies was conducted by two independent reviewers (A.M. and J.B.P.), with a third independent reviewer resolving any disagreements (T.C.J.). Data extraction and risk-of-bias assessment were performed by one reviewer (A.M.) and validated by a second independent reviewer (J.B.P.). Extracted variables of interest included: study authors, year of publication, country, study design, number of participants, description of cohorts included, combination antibiotic therapy regimen, standard-of-care antibiotic therapy regimen, primary outcomes definitions, number of participants, and rates of treatment success or failure, mortality, persistent bacteraemia, and adverse events requiring treatment discontinuation. Risk of bias was assessed using the revised Cochrane RoB 2 tool for RCTs.^19^

### Statistical analysis

Statistical analysis was performed using the ‘metafor’ package in R version 4.2.2 (R Core Team, Vienna, Austria) via the RStudio interface. We used random effects meta-analysis to evaluate the differences in the outcomes between combination therapy and standard-of-care therapy groups. Given the anticipated low number of studies to be included, the Knapp–Hartung adjustment was performed.^20^ When applicable, a 0.5 continuity correction was used for event rates of zero. Risk ratios (RRs) were meta-analysed for a primary outcome of treatment success or failure (per individual study definitions), and secondary outcomes of mortality, persistent bacteraemia, and adverse events leading to treatment discontinuation. Q-statistic and *I^2^* test were used to assess heterogeneity between the included studies, and influence analysis was used to identify any studies with disproportionately large effects on the results. Publication bias was assessed with funnel plots.

Additionally, we performed a post hoc Bayesian meta-analysis using the ‘bayesmeta’ package, with a non-informative prior probability distribution.

Finally, we evaluated the strength of the evidence using the Evidence-based Practice Center model of the US Agency for Healthcare Research and Quality.^21^

## Results

**Figure 1.**
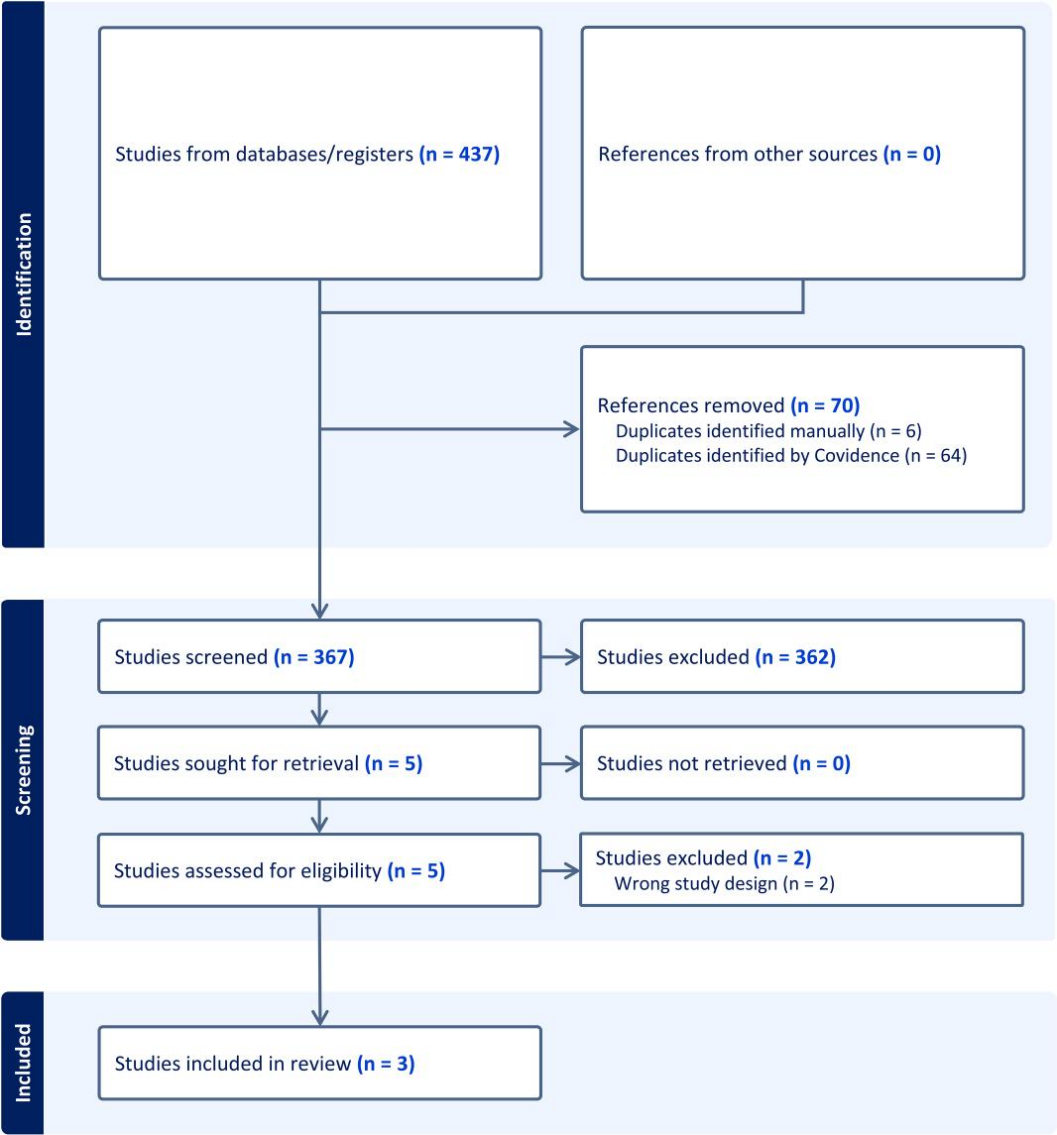
PRISMA flowchart of screened and included studies.

Our search identified a total of 437 articles. After deduplication, 367 were included for title and abstract screening (Figure 1). Five studies were included in full-text review, and three met all four prespecified eligibility criteria for inclusion in our systematic review and meta-analysis.^13,14,22^ Table S3 details the reasons for exclusion of the two articles in full-text review. The characteristics of the included RCTs are detailed in Table 1. All three included RCTs were randomized, open-label, multicentre studies, conducted in Spain; and evaluated treatment success or cure, persistent bacteraemia at 7 days, mortality and adverse events leading to treatment discontinuation. Two studies were terminated early before reaching the planned enrolment sample size. The Pericàs *et al.* study^22^ was terminated for operational futility after difficulties with enrolment, and the Grillo *et al.* study^14^ was terminated early for clinical futility at the prespecified interim analysis. Only the Pericàs *et al.* study had a high risk of bias (Table S4). A total of 384 participants were randomized and had relevant outcomes measures across the three studies.

**Table 1.** Characteristics of included RCTs

Study	Year	Country	Design	Cohort	Sample size	Combination therapy arm	Standard-of-care arm	Primary outcome	Reported adverse events of therapy
Pericàs *et al.*^22,a^	2018	Spain	Randomized, multicentre, open label	Complicated MRSA bacteraemia and/or endocarditis	15 (Comb. = 8; SoC = 7)	Fosfomycin 2 g IV every 6 h plus imipenem 1 g IV every 6 h	Vancomycin IV dosed to achieve trough level ≥15 mg/L	Persistent bacteraemia at Day 7	Drug-related toxicity
Pujol *et al.*^13^	2021	Spain	Randomized, multicentre, open label	Uncomplicated and complicated MRSA bacteraemia	155^b^ (Comb. = 74; SoC = 81)	Fosfomycin 2 g IV every 6 h plus daptomycin 10 mg/kg IV once daily	Daptomycin 10 mg/kg IV once daily	Treatment success at 6 wk after end of therapy (test-of-cure): alive, resolution of clinical manifestations of infection, negative blood cultures at test-of-cure, and absence of criteria for treatment failure^c^	Adverse events leading to treatment discontinuation
Grillo *et al.*^14^	2023	Spain	Randomized, multicentre, open label	Uncomplicated and complicated MSSA bacteraemia	214^b^ (Comb. = 104; SoC = 110)	Fosfomycin 3 g IV every 6 h plus cloxacillin 2 g IV every 4 h (administered for ≥7 d after randomization)	Cloxacillin 2 g IV every 4 h (administered for ≥7 d after randomization)	Treatment success at Day 7: alive, stable or improved qSOFA score, afebrile, with negative blood cultures	Adverse events leading to treatment discontinuation in the first 7 d of therapy

Comb., combination therapy; qSOFA, quick Sequential Organ Failure Assessment; SoC, standard of care.

^a^Pericàs *et al*.^22^ defined cure from complicated MRSA bacteraemia and/or endocarditis (i.e. ‘treatment success’ for the purposes of our meta-analysis) as ‘survival and absence of relapse without change of treatment arm at 4 and 12 weeks follow-up’.

^b^Number assessed for the primary outcome.

^c^Treatment failure included any of the following: lack of clinical improvement ≥3 d after start of therapy, persistent MRSA bacteraemia ≥7 d after start of therapy, premature discontinuation of therapy due to adverse events or based on clinical judgement, recurrent MRSA bacteraemia before or at test-of-cure, additional antimicrobial therapy active against MRSA administered before test-of-cure, lack of blood cultures obtained at test-of-cure, and/or death due to any cause before test-of-cure.

### Treatment success or cure

A total of 127/186 (68.3%) participants receiving combination therapy with fosfomycin and 119/198 (60.1%) receiving standard-of-care antibiotic therapy had treatment success or cure (Table 2). However, given the significant clinical heterogeneity in definitions of treatment success or cure among the three studies, meta-analysis was deemed not appropriate and hence was not conducted.

**Table 2.** Outcomes of participants receiving combination therapy with fosfomycin versus standard-of-care antibiotic therapy

	Treatment success or cure^a^		Mortality		Persistent bacteraemia^b^		Adverse events^c^	
	Combination	SoC	Combination	SoC	Combination	SoC	Combination	SoC
Pericàs *et al.*^22^	4/8 (50%)	3/7 (42.9%)	4/8 (50%)^d^	1/7 (14.3%)^d^	0/8	1/7 (14.3%)	1/8 (12.5%)	1/7 (14.3%)
Pujol *et al.*^13^	40/74 (54.1%)	34/81 (42.0%)	18/74 (24.3%)^e^	22/81 (27.2%)^e^	0/74	5/81 (6.2%)	13/74 (17.6%)	4/81 (4.9%)
Grillo *et al.*^14^	83/104 (79.8%)	82/110 (74.5%)	10/104 (9.6%)^d^	17/110 (15.5%)^d^	2/90 (2.2%)	4/97 (4.1%)	11/104 (10.6%)	9/110 (8.2%)
**Total**	127/186 (68.3%)	119/198 (60.1%)	32/186 (17.2%)	40/198 (20.2%)	2/172 (1.2%)	10/185 (5.4%)	25/186 (13.4%)	14/198 (7.1%)

^a^See Table 1 for definitions; meta-analysis not performed on treatment success/cure.

^b^Positive blood culture at Day 7 after randomization.

^c^Adverse events leading to treatment discontinuation.

^d^Mortality 12 wk after randomization.

^e^Mortality 6 wk after completion of therapy.

### Mortality, persistent bacteraemia and adverse events

**Figure 2.**
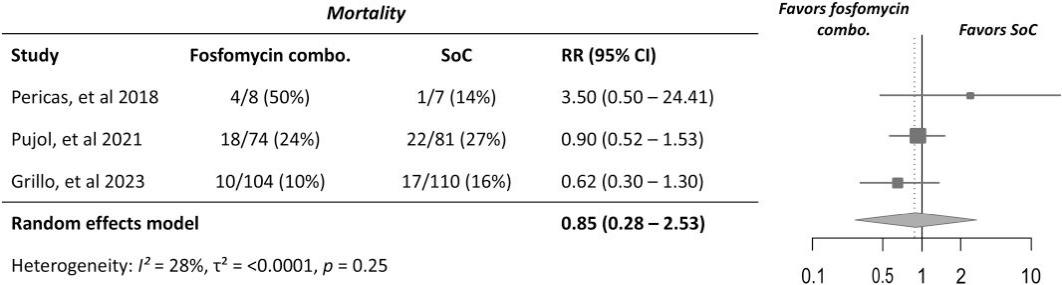
Forest plot of mortality in study participants receiving combination therapy with fosfomycin versus standard-of-care (SoC) antibiotic therapy for *S. aureus* bacteraemia or endocarditis. RR < 1 favours combination therapy group—i.e. less likely to have mortality.

A total of 32/186 (17.2%) participants receiving combination therapy with fosfomycin and 40/198 (20.2%) receiving standard-of-care antibiotic therapy died within the follow-up period (Table 2). The included studies assessed mortality at either 6 weeks after completion of therapy or 12 weeks after randomization. This endpoint was deemed appropriate for meta-analysis. There was no significant difference in mortality between combination therapy and standard-of-care groups (RR 0.85; 95% CI, 0.28–2.52; *I^2^* = 27.8%) (Figure 2). However, the CIs were wide, and there was mild to moderate heterogeneity between the studies.

**Figure 3.**
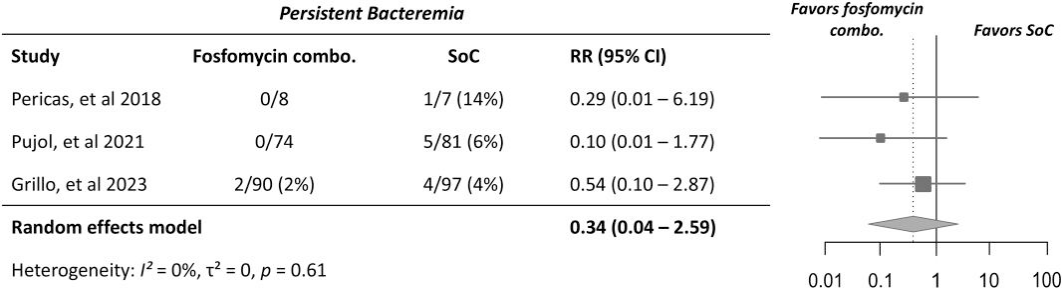
Forest plot of persistent bacteraemia in study participants receiving combination therapy with fosfomycin versus standard-of-care (SoC) antibiotic therapy for *S. aureus* bacteraemia or endocarditis. RR < 1 favours combination therapy group—i.e. less likely to have persistent bacteraemia.

Among participants assessed for persistent bacteraemia at Day 7 from randomization, a total of 2/172 (1.2%) participants receiving combination therapy with fosfomycin and 10/185 (5.4%) receiving standard-of-care antibiotic therapy had persistent bacteraemia (Table 2). Although combination therapy had a lower proportion of participants with persistent bacteraemia, there was no significant difference between these groups in meta-analysis (RR 0.34; 95% CI, 0.04–2.59; *I^2^* = 0%) (Figure 3). Heterogeneity among studies was low.

**Figure 4.**
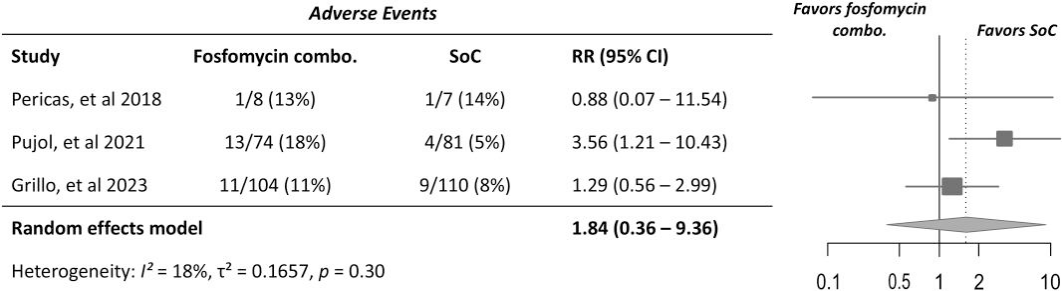
Forest plot of treatment-related adverse events leading to treatment discontinuation in study participants receiving combination therapy with fosfomycin versus standard-of-care (SoC) antibiotic therapy for *S. aureus* bacteraemia or endocarditis. RR > 1 favours SoC therapy group—i.e. combination therapy more likely to have treatment-related adverse events.

Finally, 25/186 (13.4%) participants receiving combination therapy with fosfomycin and 14/198 (7.4%) receiving standard-of-care antibiotic therapy experienced an adverse event resulting in discontinuation of therapy (Table 2). Similarly, this was not significantly different between groups in meta-analysis (RR 1.84; 95% CI, 0.36–9.36; *I^2^* = 18%) (Figure 4). There was mild to moderate heterogeneity between the studies, and the pooled CI was substantially wide, with a high upper bound.

There were no studies that significantly changed these effect estimates when excluded in influence analysis (Figures S1–S3).

### Post hoc Bayesian meta-analysis

Utilizing a non-informative prior probability distribution, post hoc Bayesian meta-analysis showed the overall OR for mortality was 0.84 (95% credible interval, 0.39–1.95) with a posterior probability for OR < 1 (i.e. combination therapy reduces mortality) of 65.5%; the OR for persistent bacteraemia was 0.31 (95% credible interval, 0.07–1.37) with a posterior probability for OR < 1 (i.e. combination therapy reduces persistent bacteraemia) of 93.8%. The OR for adverse events leading to treatment discontinuation, however, was 1.97 (95% credible interval, 0.76–5.11) with a posterior probability for OR > 1 (i.e. combination therapy increases adverse events leading to treatment discontinuation) of 92.2%.

### Evaluation of the evidence

Although this systematic review and meta-analysis only included RCTs of relatively similar populations, given the early termination of two of three of the studies, and the low sample size introducing concerns with the selection of reporting of results in the Pericàs *et al.* study, the strength of evidence was downgraded to moderate (Table S5). Publication bias analysis is provided in Figure S4.

## Discussion

In our meta-analysis of three RCTs that included 384 participants, we sought to answer the question ‘for patients with bacteraemia or endocarditis caused by *S. aureus*, what is the efficacy and safety of combination therapy with fosfomycin compared with standard-of-care antibiotic therapy?’ We identified three key findings: (i) combination therapy with fosfomycin did not significantly improve mortality in participants with *S. aureus* bacteraemia or endocarditis; (ii) combination therapy may reduce rates of persistent *S. aureus* bacteraemia; however, the difference was not statistically significant, and the CIs were wide; and (iii) combination therapy may increase the rate of adverse events leading to treatment discontinuation; however, this was also not statistically significant and the CIs were wide.

Combination, or adjunctive, antibiotic therapy regimens for routine management of *S. aureus* bacteraemia or endocarditis have been evaluated in robust randomized clinical trials with varying levels of success.^8^ Most notably, a combination therapy regimen of vancomycin or daptomycin with or without an anti-staphylococcal β-lactam in patients with MRSA bacteraemia decreased the rate of persistent bacteraemia at 5 days (6% versus 11%, *P* < 0.001), but the rate of acute kidney injury was almost 4-fold higher (23% versus 6%, *P* < 0.001).^23^ Furthermore, there was no difference in 90 day mortality between the groups. The addition of rifampicin to standard-of-care antibiotic therapy was also evaluated in a large RCT including patients with both MSSA and MRSA bacteraemia, and no significant difference in treatment failure was found between the groups. Similarly, participants receiving adjunctive rifampicin were more likely to experience adverse events—in this case, requiring modification to antibiotic regimens (17% versus 10%).^24^ For prosthetic valve staphylococcal endocarditis, adjunctive therapy with an aminoglycoside and/or rifampicin has been traditionally recommended by practice guidelines. However, a recent meta-analysis of over 300 patients found that combination therapy with either or both agents did not reduce the risk of treatment failure and once again was more likely to result in adverse events (approximately one-third of patients).^25^

Our meta-analysis suggests that combination therapy with fosfomycin may be subject to the same limitations as other combination regimens previously studied—it may shorten the duration of bacteraemia but at the cost of increased treatment-related adverse events without improving patient-centred clinical outcomes such as treatment failure or mortality. The wide CIs, even when the studies were pooled, highlight the degree of uncertainty in estimating these effects. Despite not meeting statistical significance, the high upper bound of the CI for treatment-related adverse events would suggest that there is a potential for a higher rate of adverse events among the combination therapy group.

Although the included RCTs had low heterogeneity in the conducted meta-analyses, there were some important differences in study design that limited our ability to analyse some of the data, i.e. treatment success or cure. For example, Grillo *et al.*^14^ reported significantly higher treatment success and lower mortality rates compared with the other studies. This is likely because clinical treatment success was measured at Day 7, rather than several weeks after randomization or completion of treatment. We could not extract longer-term treatment success rates from the available data, although the authors do note that 21/95 (22.1%) participants in the combination therapy group and 35/105 (33.3%) in the standard-of-care group had complicated bacteraemia at test-of-cure. These results suggest that treatment success at Day 7 underestimates *S. aureus*–related complications that can increase the likelihood of treatment failure over time.

An important consideration in the design of clinical trials for *S. aureus* bacteraemia or endocarditis is how treatment success or failure are defined. In some instances, treatment failure may be attributed to incomplete study procedures that may have no bearing on the *clinical* success or failure of the participant in the trial. For example, participants may be categorized as experiencing treatment failure if blood cultures are not obtained at the test-of-cure visit. Although test-of-cure blood cultures are commonly required by regulatory bodies for Phase 3 registrational trials,^26,27^ there is a very low risk of unrecognized *S. aureus* bacteraemia in a patient who is clinically well weeks after completion of antibiotic therapy. A thought experiment demonstrates the potential implications of these administrative failures: in the Pujol *et al.* study,^13^ in which individual participants could have multiple reasons for treatment failure, the authors report that 8/34 (23.5%) participants in the combination therapy group and 4/47 (8.5%) in the standard-of-care group did not have blood cultures obtained at test-of-cure. If we assume that these participants did not have any other criteria for treatment failure, and otherwise met criteria for treatment success, then the likelihood of treatment success with combination therapy in this study would increase to a statistically significant RR of 1.38 (95% CI, 1.04–1.84). This demonstrates that careful attention to outcome definitions may improve pragmatism and minimize likelihood of type II error; and the use of a patient-centred outcome may minimize the impact of administrative failures on the assessment of efficacy.

The pooled estimates of effect sizes in this study may still help inform the design of future studies of combination therapy. The ongoing *Staphylococcus aureus* Network Adaptive Platform (SNAP),^28^ the largest randomized clinical trial of patients with *S. aureus* bacteraemia therapy to date, may evaluate adjunctive fosfomycin for the treatment of *S. aureus* bacteraemia.^29^ Given the very large sample size, SNAP could provide more robust estimates of the efficacy and safety of combination therapy with fosfomycin. One important consideration, however, is that IV fosfomycin formulations are currently not available in all countries, including the USA—which may limit global generalizability and implementation.

### Limitations

Our study has at least three limitations. First, although our stringent eligibility criteria and *a priori* decision to only include RCTs served to minimize bias and confounding from observational studies, the result was a small number of studies included. Second, given the relatively small sample size after meta-analysis, we were unable to perform sub-group analysis to evaluate the effect of combination therapy on the outcomes of specific patient groups (i.e. MSSA versus MRSA). Finally, even among clinical trials conducted among similar patient groups within the same country, differences in study design (i.e. duration of follow-up for the relevant outcome) may complicate interpretation of meta-analyses.

## Conclusion

In this systematic review and meta-analysis of three randomized trials evaluating the efficacy and safety of combination therapy with fosfomycin compared with standard-of-care antibiotic therapy for *S. aureus* bacteraemia or endocarditis, combination therapy did not significantly improve patient outcomes and may be associated with higher rates of treatment-related adverse events. Based on these findings, there is likely no role, at present, for routine combination therapy with fosfomycin for *S. aureus* bacteraemia or endocarditis. However, given a favourable point estimate for mortality, and persistent bacteraemia (and high posterior probability for success in post hoc Bayesian meta-analysis), but wide CIs, larger trials may be needed to better understand if fosfomycin combination therapy may ultimately improve the care of this complex patient population.

## Supplementary Material

dlaf101_Supplementary_Data

## Supplementary data

Figures S1 to S4 and Tables S1 to S5 are available as Supplementary data at *JAC-AMR* Online.
