# Supplementary material for: Combination therapy with fosfomycin for Staphylococcus aureus bacteraemia or endocarditis: a systematic review and meta-analysis of randomized trials
Source: JAC Antimicrob Resist. 2025 Jun 17;7(3):dlaf101. doi: 10.1093/jacamr/dlaf101 (PMC12199917; doi:10.1093/jacamr/dlaf101)
Supplement: dlaf101_Supplementary_Data [file dlaf101_supplementary_data.docx]

**Supplementary Appendix**

| **1** | PRISMA-SR checklist | Page 2-4 |
| --- | --- | --- |
| **2** | Search strategy | Page 5-8 |
| **3** | List of excluded studies | Page 9 |
| **4** | Risk of bias assessment | Page 10 |
| **5** | Influence analysis for mortality | Page 11 |
| **6** | Influence analysis for persistent bacteremia | Page 12 |
| **7** | Influence analysis for adverse events | Page 13 |
| **8** | Publication bias analysis | Page 14 |
| **9** | Strength of evidence grading | Page 15 |

**Table S1.** PRISMA-SR Checklist

| **Section and Topic** | **Item #** | **Checklist item** | **Location where item is reported** |
| --- | --- | --- | --- |
| **TITLE** | | | |
| Title | 1 | Identify the report as a systematic review. | 1 |
| **ABSTRACT** | | | |
| Abstract | 2 | See the PRISMA 2020 for Abstracts checklist. | 2 |
| **INTRODUCTION** | | | |
| Rationale | 3 | Describe the rationale for the review in the context of existing knowledge. | 3 |
| Objectives | 4 | Provide an explicit statement of the objective(s) or question(s) the review addresses. | 3 |
| **METHODS** | | | |
| Eligibility criteria | 5 | Specify the inclusion and exclusion criteria for the review and how studies were grouped for the syntheses. | 4-5 |
| Information sources | 6 | Specify all databases, registers, websites, organisations, reference lists and other sources searched or consulted to identify studies. Specify the date when each source was last searched or consulted. | 4 |
| Search strategy | 7 | Present the full search strategies for all databases, registers and websites, including any filters and limits used. | Supplementary table 2 |
| Selection process | 8 | Specify the methods used to decide whether a study met the inclusion criteria of the review, including how many reviewers screened each record and each report retrieved, whether they worked independently, and if applicable, details of automation tools used in the process. | 5 |
| Data collection process | 9 | Specify the methods used to collect data from reports, including how many reviewers collected data from each report, whether they worked independently, any processes for obtaining or confirming data from study investigators, and if applicable, details of automation tools used in the process. | 5 |
| Data items | 10a | List and define all outcomes for which data were sought. Specify whether all results that were compatible with each outcome domain in each study were sought (e.g. for all measures, time points, analyses), and if not, the methods used to decide which results to collect. | 5 |
|  | 10b | List and define all other variables for which data were sought (e.g. participant and intervention characteristics, funding sources). Describe any assumptions made about any missing or unclear information. | 5 |
| Study risk of bias assessment | 11 | Specify the methods used to assess risk of bias in the included studies, including details of the tool(s) used, how many reviewers assessed each study and whether they worked independently, and if applicable, details of automation tools used in the process. | 5 |
| Effect measures | 12 | Specify for each outcome the effect measure(s) (e.g. risk ratio, mean difference) used in the synthesis or presentation of results. | 5-6 |
| Synthesis methods | 13a | Describe the processes used to decide which studies were eligible for each synthesis (e.g. tabulating the study intervention characteristics and comparing against the planned groups for each synthesis (item #5)). | 5-6 |
|  | 13b | Describe any methods required to prepare the data for presentation or synthesis, such as handling of missing summary statistics, or data conversions. | 5-6 |
|  | 13c | Describe any methods used to tabulate or visually display results of individual studies and syntheses. | 5-6 |
|  | 13d | Describe any methods used to synthesize results and provide a rationale for the choice(s). If meta-analysis was performed, describe the model(s), method(s) to identify the presence and extent of statistical heterogeneity, and software package(s) used. | 6 |
|  | 13e | Describe any methods used to explore possible causes of heterogeneity among study results (e.g. subgroup analysis, meta-regression). | 6 |
|  | 13f | Describe any sensitivity analyses conducted to assess robustness of the synthesized results. | 6 |
| Reporting bias assessment | 14 | Describe any methods used to assess risk of bias due to missing results in a synthesis (arising from reporting biases). | 9 |
| Certainty assessment | 15 | Describe any methods used to assess certainty (or confidence) in the body of evidence for an outcome. | 5 |
| **RESULTS** | | | |
| Study selection | 16a | Describe the results of the search and selection process, from the number of records identified in the search to the number of studies included in the review, ideally using a flow diagram. | 6, Figure 1 |
|  | 16b | Cite studies that might appear to meet the inclusion criteria, but which were excluded, and explain why they were excluded. | Supplementary Table 3 |
| Study characteristics | 17 | Cite each included study and present its characteristics. | Table 1 |
| Risk of bias in studies | 18 | Present assessments of risk of bias for each included study. | Supplementary Table 4 |
| Results of individual studies | 19 | For all outcomes, present, for each study: (a) summary statistics for each group (where appropriate) and (b) an effect estimate and its precision (e.g. confidence/credible interval), ideally using structured tables or plots. | Table 2, Figure 2 |
| Results of syntheses | 20a | For each synthesis, briefly summarise the characteristics and risk of bias among contributing studies. | Supplementary Table 4 |
|  | 20b | Present results of all statistical syntheses conducted. If meta-analysis was done, present for each the summary estimate and its precision (e.g. confidence/credible interval) and measures of statistical heterogeneity. If comparing groups, describe the direction of the effect. | Figure 2  Figure 3  Figure 4  Figure 5 |
|  | 20c | Present results of all investigations of possible causes of heterogeneity among study results. | 7-9 |
|  | 20d | Present results of all sensitivity analyses conducted to assess the robustness of the synthesized results. | 7-9, Supplementary Figure 1 |
| Reporting biases | 21 | Present assessments of risk of bias due to missing results (arising from reporting biases) for each synthesis assessed. | NA |
| Certainty of evidence | 22 | Present assessments of certainty (or confidence) in the body of evidence for each outcome assessed. | Figures 2, 3, 4, 5 |
| **DISCUSSION** | | | |
| Discussion | 23a | Provide a general interpretation of the results in the context of other evidence. | 9 |
|  | 23b | Discuss any limitations of the evidence included in the review. | 12 |
|  | 23c | Discuss any limitations of the review processes used. | 12 |
|  | 23d | Discuss implications of the results for practice, policy, and future research. | 9-13 |
| **OTHER INFORMATION** | | | |
| Registration and protocol | 24a | Provide registration information for the review, including register name and registration number, or state that the review was not registered. | 4 |
|  | 24b | Indicate where the review protocol can be accessed, or state that a protocol was not prepared. | 4 |
|  | 24c | Describe and explain any amendments to information provided at registration or in the protocol. | N/A |
| Support | 25 | Describe sources of financial or non-financial support for the review, and the role of the funders or sponsors in the review. | 14 |
| Competing interests | 26 | Declare any competing interests of review authors. | 14 |
| Availability of data, code and other materials | 27 | Report which of the following are publicly available and where they can be found: template data collection forms; data extracted from included studies; data used for all analyses; analytic code; any other materials used in the review. | N/A (data is publicly available) |

**Table S2.** Search strategy.

| **Database (including vendor/platform):** PubMed MEDLINE  **Search Date:** 9/12/2024 | | | |
| --- | --- | --- | --- |
| **Search step** | **Search string** | **Results** |  |
| 1. S. aureus | "Staphylococcus aureus"[Mesh] OR "staphylococcus aureus"[tiab] OR "s. aureus"[tiab] OR "s aureus"[tiab] OR "staph aureus"[tiab] OR Staphylococcusaureus[tiab] OR "Methicillin-Resistant Staphylococcus aureus"[Mesh] OR ("Methicillin Resistance"[Mesh] AND "Staphylococcal Infections"[Mesh]) OR mrsa[tiab] OR mssa[tiab] | 165,730 |  |
| 1. Bacteremia | "Bacteremia"[Mesh] OR Bacteremia[tiab] OR bacteriaemia[tiab] OR Bacteraemia[tiab] OR Bacteremias[tiab] OR bacteriaemias[tiab] OR Bacteraemias[tiab] OR bacteremic[tiab] OR bacteraemic[tiab] OR ((bloodstream[tiab] OR "blood stream"[tiab] OR bloodstreams[tiab] OR "blood streams"[tiab]) AND ("Infections"[Mesh] OR infection[tiab] OR infections[tiab] OR infected[tiab] OR infect[tiab] OR infects[tiab] OR infecting[tiab])) | 72,634 |  |
| 1. endocarditis | “Endocarditis"[Mesh] OR Endocarditis[tiab] OR Endocarditides[tiab] OR (("endocardium"[Mesh] OR endocardium[tiab]) AND ("Inflammation"[Mesh] OR inflammation[tiab] OR inflammations[tiab] OR inflamed[tiab] OR inflammatory[tiab])) | 47,599 |  |
| 1. bacteremia OR endocarditis | #2 OR #3 | 115,315 |  |
| 1. fosfomycin | "Fosfomycin"[Mesh] OR Fosfomycin[tiab] OR Fosfomycinum[tiab] OR Fosfomycine[tiab] OR Phosphomycin[tiab] OR Phosphonomycin[tiab] OR Phosphonemycin[tiab] OR Fosfomycinum[tiab] OR Monuril[tiab] OR Monurol[tiab] OR Fosfocina[tiab] OR Fosfomicina[tiab] | 4,814 |  |
| 1. Standard RCT filter (DMCL adapted version of Cochrane Highly Sensitive Strategy hybrid) | "randomized controlled trial"[pt] OR "controlled clinical trial"[pt] OR "drug therapy"[sh] OR randomized[tiab] OR randomised[tiab] OR randomization[tiab] OR randomisation[tiab] OR randomly[tiab] OR placebo[tiab] OR trial[tiab] OR groups[tiab] | 6,185,885 |  |
| 1. S. aureus + (bacteremia OR endocarditis) + fosfomycin + sensitive Cochrane RCT filter | #1 AND #4 AND #5 AND #6 | 66 |  |
| 1. Human filter | #7 NOT ("Animals"[mh] NOT "Humans"[mh]) | 59 |  |
| 1. Exemplar articles/search validation | 32725216 OR 37783969 OR 25048851 OR 29408610 | 4/4 retrieved |  |

| **Database (including vendor/platform):** Embase (Elsevier)  **Search Date:** 9/12/2024 | | |
| --- | --- | --- |
| **Search step** | **Search string** | **Results** |
| 1. S. aureus | 'staphylococcus aureus'/exp OR 'staphylococcus aureus':ti,ab OR 's. aureus':ti,ab OR 's aureus':ti,ab OR 'staph aureus':ti,ab OR staphylococcusaureus:ti,ab OR 'Methicillin-Resistant Staphylococcus aureus'/exp OR ('Methicillin Resistance'/exp AND 'Staphylococcal Infections'/exp) OR mrsa:ti,ab OR mssa:ti,ab | 276,522 |
| 1. Bacteremia | 'bacteremia'/exp OR Bacteremia:ti,ab OR bacteriaemia:ti,ab OR Bacteraemia:ti,ab OR Bacteremias:ti,ab OR bacteriaemias:ti,ab OR Bacteraemias:ti,ab OR bacteremic:ti,ab OR bacteraemic:ti,ab OR ((bloodstream:ti,ab OR 'blood stream':ti,ab OR bloodstreams:ti,ab OR 'blood streams':ti,ab) AND (Infections/exp OR infection:ti,ab OR infections:ti,ab OR infected:ti,ab OR infect:ti,ab OR infects:ti,ab OR infecting:ti,ab)) | 110,308 |
| 1. endocarditis | Endocarditis/exp OR Endocarditis:ti,ab OR Endocarditides:ti,ab OR ((endocardium/exp OR endocardium:ti,ab) AND (Inflammation/exp OR inflammation:ti,ab OR inflammations:ti,ab OR inflamed:ti,ab OR inflammatory:ti,ab)) | 78,512 |
| 1. bacteremia OR endocarditis | #2 OR #3 | 179,066 |
| 1. fosfomycin | 'fosfomycin'/exp OR fosfomycin:ti,ab OR fosfomycine:ti,ab OR phosphomycin:ti,ab OR phosphonomycin:ti,ab OR phosphonemycin:ti,ab OR fosfomycinum:ti,ab OR monuril:ti,ab OR monurol:ti,ab OR fosfocina:ti,ab OR fosfomicina:ti,ab | 15,248 |
| 1. Standard RCT filter (DMCL adapted version of Cochrane Highly Sensitive Strategy hybrid) | 'randomized controlled trial'/exp OR 'controlled clinical trial'/exp OR 'drug therapy'/exp OR randomized:ab,ti OR randomised:ab,ti OR randomization:ab,ti OR randomisation:ab,ti OR randomly:ab,ti OR placebo:ti,ab OR trial:ti,ab OR groups:ti,ab | 8,733,729 |
| 1. S. aureus + (bacteremia OR endocarditis) + fosfomycin + sensitive Cochrane RCT filter | #1 AND #4 AND #5 AND #6 | 254 |
| 1. Human filter | #7 NOT ([animals]/lim NOT [humans]/lim) | 245 |
| 1. Exemplar articles/search validation | 32725216 OR 37783969 OR 25048851 OR 29408610 | 4/4 retrieved |

| **Database (including vendor/platform):** Cochrane Library  **Search Date:** 9/12/2024 | | |
| --- | --- | --- |
| **Search step** | **Search string** | **Results** |
| 1. S. aureus | [mh "Staphylococcus aureus"] OR "staphylococcus aureus":ti,ab OR "s. aureus":ti,ab OR "s aureus":ti,ab OR "staph aureus":ti,ab OR Staphylococcusaureus:ti,ab OR [mh "Methicillin-Resistant Staphylococcus aureus"] OR ([mh "Methicillin Resistance"] AND [mh "Staphylococcal Infections"]) OR mrsa:ti,ab OR mssa:ti,ab | 4071 |
| 1. Bacteremia | [mh Bacteremia] OR Bacteremia:ti,ab OR bacteriaemia:ti,ab OR Bacteraemia:ti,ab OR Bacteremias:ti,ab OR bacteriaemias:ti,ab OR Bacteraemias:ti,ab OR bacteremic:ti,ab OR bacteraemic:ti,ab OR ((bloodstream:ti,ab OR "blood stream":ti,ab OR bloodstreams:ti,ab OR "blood streams":ti,ab) AND (infection:ti,ab OR infections:ti,ab OR infected:ti,ab OR infect:ti,ab OR infects:ti,ab OR infecting:ti,ab)) | 4426 |
| 1. endocarditis | [mh Endocarditis] OR Endocarditis:ti,ab OR Endocarditides:ti,ab OR (([mh endocardium] OR endocardium:ti,ab) AND ([mh Inflammation] OR inflammation:ti,ab OR inflammations:ti,ab OR inflamed:ti,ab OR inflammatory:ti,ab)) | 734 |
| 1. bacteremia OR endocarditis | #2 OR #3 | 4984 |
| 1. fosfomycin | [mh fosfomycin] OR fosfomycin:ti,ab OR fosfomycine:ti,ab OR phosphomycin:ti,ab OR phosphonomycin:ti,ab OR phosphonemycin:ti,ab OR fosfomycinum:ti,ab OR monuril:ti,ab OR monurol:ti,ab OR fosfocina:ti,ab OR fosfomicina:ti,ab | 389 |
| 1. S. aureus + (bacteremia OR endocarditis) + fosfomycin + sensitive Cochrane RCT filter | #1 AND #4 AND #5 | 17 |

| **Database (including vendor/platform):** Web of Science (Clarivate) [Web of Science Core Collection, All Editions]  **Search Date:** 9/12/2024 | | |
| --- | --- | --- |
| **Search step** | **Search string** | **Results** |
| 1. S. aureus | TS=("staphylococcus aureus" OR "s. aureus" OR "s aureus" OR "staph aureus" OR Staphylococcusaureus OR mrsa OR mssa) | 213,813 |
| 1. Bacteremia | TS=(Bacteremia OR bacteriaemia OR Bacteraemia OR Bacteremias OR bacteriaemias OR Bacteraemias OR bacteremic OR bacteraemic) OR TS=((bloodstream OR "blood stream" OR bloodstreams OR "blood streams") AND (infection OR infections OR infected OR infect OR infects OR infecting)) | 75,770 |
| 1. endocarditis | TS=(Endocarditis OR Endocarditides) OR TS= ((endocardium OR endocardiums) AND (inflammation OR inflammations OR inflamed OR inflammatory)) | 44,555 |
| 1. bacteremia OR endocarditis | #2 OR #3 | 114,303 |
| 1. fosfomycin | TS=(fosfomycin OR fosfomycine OR phosphomycin OR phosphonomycin OR phosphonemycin OR fosfomycinum OR monuril OR monurol OR fosfocina OR fosfomicina) | 4,784 |
| 1. S. aureus + (bacteremia OR endocarditis) + fosfomycin + sensitive Cochrane RCT filter | #1 AND #4 AND #5 | 116 |

**Table S3.** Reasons for exclusion of studies in full text review

| **#** | **Study author, year** | **Reason for exclusion** |
| --- | --- | --- |
| **1** | del Río A, Gasch O, Moreno A, et al. Efficacy and safety of fosfomycin plus imipenem as rescue therapy for complicated bacteremia and endocarditis due to methicillin-resistant Staphylococcus aureus: a multicenter clinical trial. *Clin Infect Dis*. 2014;59(8):1105-1112. doi:10.1093/cid/ciu580 | Wrong study design; single arm, no standard of care comparator group |
| **2** | Pattharachayakul, S, Na-Thalang, K, Neuhauser, M, et al. Fosfomycin (FOS) for the treatment of methicillin-resistant Staphylococcus aureus (MRSA) blood stream infections in a teaching hospital in Thailand. *Clin Infect Dis*. 2001;33(7):1103-1103 | Wrong study design; retrospective cohort study  *Note: this study was a conference abstract. However, based on the minimal amount of information available at the title/abstract screening stage, a full text review was necessary to make this determination.* |

**Table S4.** Risk of bias assessment of included studies (per Cochrane RoB2 tool)

| **Study** | **Randomization** | **Deviations from intended interventions** | **Missing outcome data** | **Measurement of the outcome** | **Selection of the reported result** | **Overall risk of bias** |
| --- | --- | --- | --- | --- | --- | --- |
| Pericàs, et al, 2018 | Some concern | Some concern | High risk | Some concern | Some concern | High risk |
| Pujol, et al, 2021 | Low concern | Low concern | Low concern | Low concern | Low concern | Low |
| Grillo, et al, 2023 | Low concern | Low concern | Low concern | Low concern | Low concern | Low |

**Figure S1.** Influence analysis of mortality.


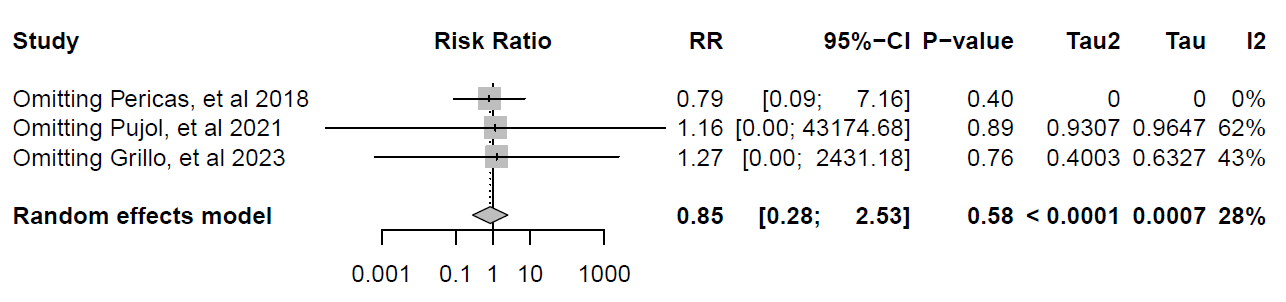


**Figure S2.** Influence analysis of persistent bacteremia.


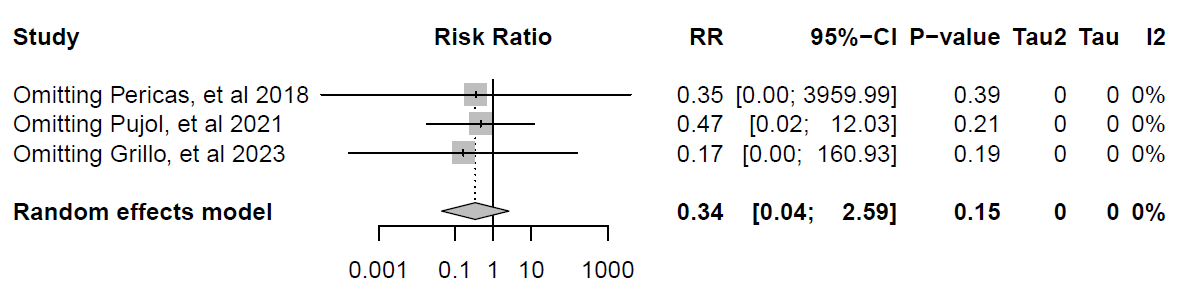


**Figure S3.** Influence analysis of adverse events.


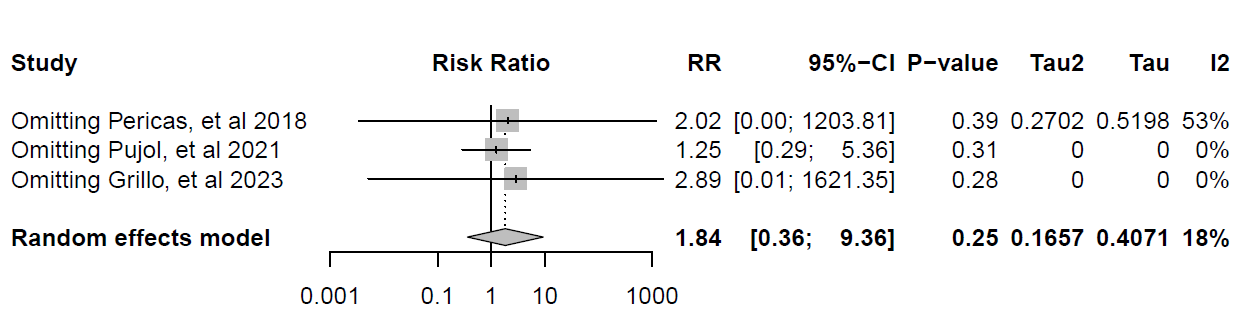


**Figure S4.** Publication bias analysis (Funnel plot – Trim and Fill method; adjusted for small number of studies).


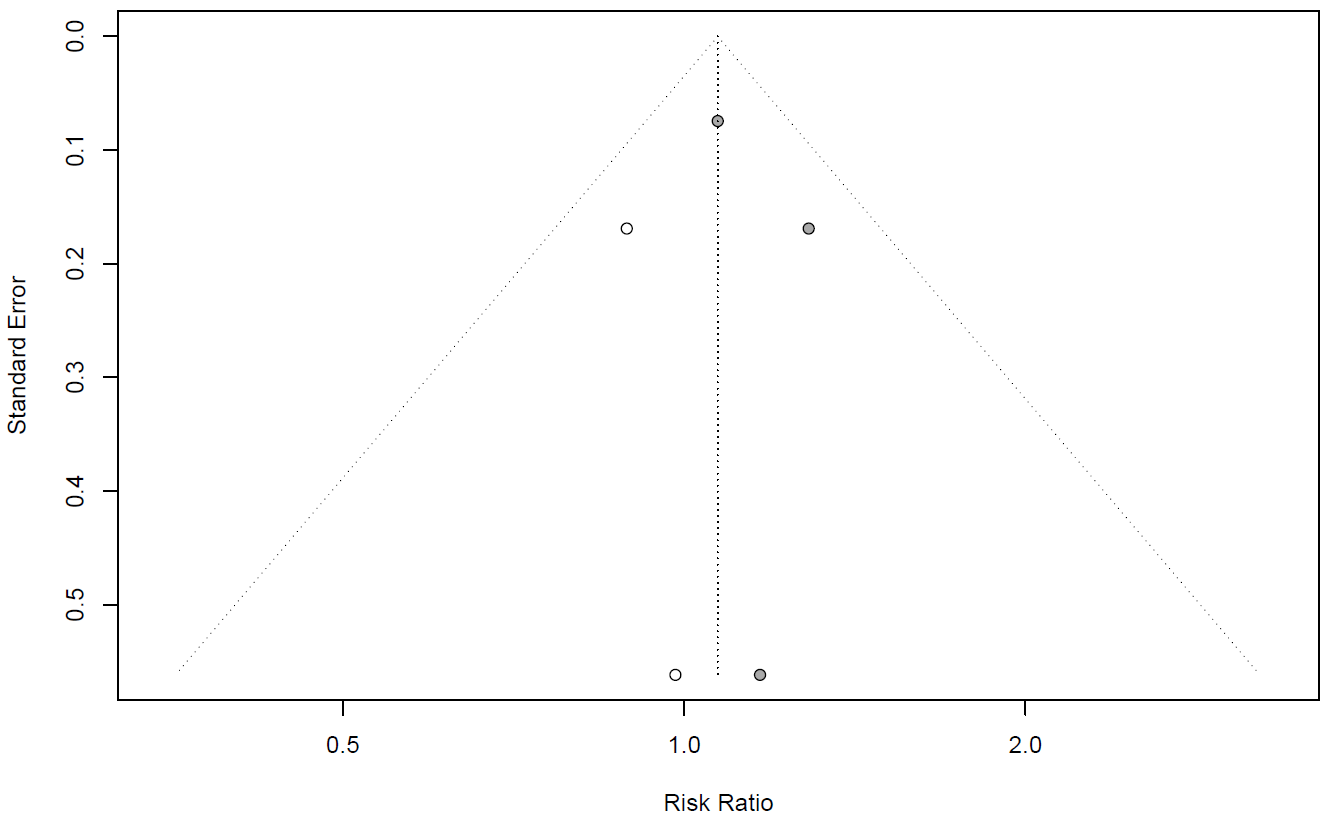


**Supplementary Table 5.** EPC – AHRQ strength of evidence grading.

| **Patient population** | Patients with bacteremia or endocarditis caused by *Staphylococcus aureus* | | | |
| --- | --- | --- | --- | --- |
| **Setting** | Hospital | | | |
| **Intervention** | Combination antibiotic therapy with fosfomycin | | | |
| **Comparator** | Standard of care antibiotic therapy | | | |
| **Outcome** | **Treatment success** | **Persistent bacteremia** | **Mortality** | **Adverse events** |
| **Studies (Participants)** | - | 3 RCTs (357) | 3 RCTs (384) | 3 RCTs (384) |
| **Risk of Bias** | - | Some concern | Some concern | Some concern |
| **Consistency** | - | Consistent | Consistent | Consistent |
| **Precision** | - | Precise | Precise | Precise |
| **Directness** | - | Direct | Direct | Direct |
| **Overall Strength of Evidence** | - | Moderate | Moderate | Moderate |
| **Conclusions** | Given the significant clinical heterogeneity in definitions of treatment success or cure among the three studies, meta-analysis was deemed not appropriate and as such not conducted. | There is likely no difference in persistent bacteremia in participants receiving combination antibiotic therapy with fosfomycin versus standard of care therapy for *S. aureus* bacteremia or endocarditis (RR 0.34; 95% CI, 0.04 - 2.59; *I^2^* = 0%) | There is likely no difference in mortality in participants receiving combination antibiotic therapy with fosfomycin versus standard of care therapy for *S. aureus* bacteremia or endocarditis (RR 0.85; 95% CI, 0.28 - 2.52; *I^2^* = 27.8%) | There is likely no difference in rate of adverse events in participants receiving combination antibiotic therapy with fosfomycin versus standard of care therapy for *S. aureus* bacteremia or endocarditis (RR 1.84; 95% CI, 0.36 - 9.36; *I^2^* = 18%). However, the wide confidence interval with a high upper bound would suggest a potential higher rate of adverse events among the combination therapy group. |
